# Extraction, Purification, Structural Characteristics, Health Benefits, and Application of the Polysaccharides from *Lonicera japonica* Thunb.: A Review

**DOI:** 10.3390/molecules28124828

**Published:** 2023-06-17

**Authors:** Xinpeng Yang, Aiqi Yu, Wenjing Hu, Zhaojiong Zhang, Ye Ruan, Haixue Kuang, Meng Wang

**Affiliations:** Key Laboratory of Basic and Application Research of Beiyao (Ministry of Education), Heilongjiang University of Chinese Medicine, The Second Affiliated Hospital of Heilongjiang University of Chinese Medicine, Harbin 150000, China

**Keywords:** *Lonicera japonica* Thunb., polysaccharide, structural characteristic, health benefits, structure–activity relationship, application

## Abstract

*Lonicera japonica* Thunb. is a widely distributed plant with ornamental, economic, edible, and medicinal values. *L. japonica* is a phytoantibiotic with broad-spectrum antibacterial activity and a potent therapeutic effect on various infectious diseases. The anti-diabetic, anti-Alzheimer’s disease, anti-depression, antioxidative, immunoregulatory, anti-tumor, anti-inflammatory, anti-allergic, anti-gout, and anti-alcohol-addiction effects of *L. japonica* can also be explained by bioactive polysaccharides isolated from this plant. Several researchers have determined the molecular weight, chemical structure, and monosaccharide composition and ratio of *L. japonica* polysaccharides by water extraction and alcohol precipitation, enzyme-assisted extraction (EAE) and chromatography. This article searched in the Chinese Pharmacopoeia, Flora of China, Web of Science, PubMed, and CNKI databases within the last 12 years, using “*Lonicera. japonica* polysaccharides”, “*Lonicera. japonica* Thunb. polysaccharides”, and “Honeysuckle polysaccharides” as the key word, systematically reviewed the extraction and purification methods, structural characteristics, structure-activity relationship, and health benefits of *L. japonica* polysaccharides to provide insights for future studies. Further, we elaborated on the potential applications of *L. japonica* polysaccharides in the food, medicine, and daily chemical industry, such as using *L. japonica* as raw material to make lozenges, soy sauce and toothpaste, etc. This review will be a useful reference for the further optimization of functional products developed from *L. japonica* polysaccharides.

## 1. Introduction

**Figure 1:**
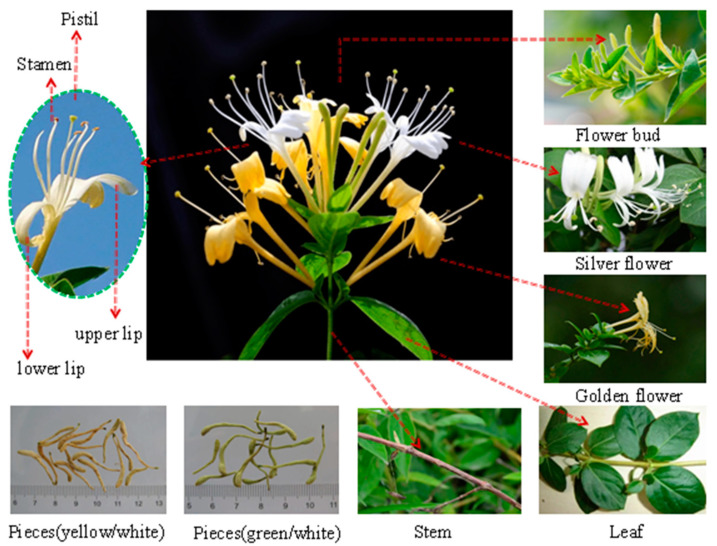
A plant image of *Lonicera japonica* Thunb. (*L. japonica*).

*Lonicera japonica* Thunb. is a common perennial semievergreen vine belonging to the genus *Lonicera* of the family Caprifoliaceae [1]. Its flower shows two colors, yellow and white, and is commonly called double flower, two flower, or mandarin duck vine (vernacular names). This phenomenon is attributed to the easy oxidation and structural instability of flavonoids, inositols, saponins, and other components, specifically anthocyanin, which gradually turn yellow after light exposure or at flower maturity. *L. japonica* flower is fragrant and blossoms in summer, and its pistil and style extend out of the corolla. The beginning of the flowering period is indicated by the appearance of two colors of flowers [2]. The flower or bud in this state has edible, nutritional, and medicinal values. A plant image of *Lonicera japonica* is shown in Figure 1. In addition, *L. japonica* can adapt and survive in adverse natural environments, including waterlogging. This characteristic allows for the worldwide distribution of the species, and it mainly grows in temperate and tropical regions in Asia, Europe and Africa [3]. *L. japonica* is a valuable cash crop that is extensively distributed throughout China. The plant has been used for various purposes and has a long history of use as a traditional Chinese medicine (TCM) [4,5,6]. The nutritional and medicinal properties of *L. japonica* have gained more attention from health-seeking consumers, nutritionists, and natural botanists.

*L. japonica* has been cultivated and consumed for over 1000 years as a nutritional supplement and for treating infections [7]. Notably, it has a broad-spectrum antibacterial activity and is considered a phytoantibiotic for treating infectious diseases [8]. In addition, *L. japonica* is an important natural antiviral in the empirical medicine system (plant prevention needle for treating viral infections) [9]. *L. japonica* is the main raw material of the two patented Chinese medicines, Lianhua Qingwen and Shuanghuanglian, which have been approved for the treatment and prevention of COVID-19 worldwide [10,11,12]. The plant has been extensively studied worldwide because of its medicinal uses. *L. japonica* is a rich source of various bioactive substances, including small-molecule compounds and macromolecular polysaccharides. Several researchers have isolated and purified small-molecule bioactive compounds from *L. japonica*, including essential oils, saponins, organic acids, iridoids, and flavonoids [13]. The polysaccharides obtained from *L. japonica* have advanced structures and unique biological properties, including anti-diabetic, anti-Alzheimer’s disease, anti-depression, anti-tumor, anti-inflammatory, anti-allergic, and anti-gout effects [14,15,16]. Moreover, as *L. japonica* polysaccharides are natural, safe, and minimally toxic with few side effects, they have several prospective uses as a health food, functional food, nutritional supplement, and as medicine.

*L. japonica* was included in the list of items that are both food and medicine by the National Health Commission of the People’s Republic of China in 2002 [17]. Further, it has been recognized for its edible, nutritional, and pharmaceutical properties with immense health benefits. *L. japonica* was also included in the Chinese Pharmacopoeia (1963–2020 edition) [18]. Further, *L. japonica* is an indispensable TCM for the clinical treatment of influenza, fever, headache, and pharyngitis [19]. Conventionally, the bioactive small-molecule compounds in *L. japonica* were considered active ingredients [20]. However, several polysaccharides have been isolated from *L. japonica* in recent years, and their diverse structure and unique biological activities are being explored worldwide [21,22]. *L. japonica* polysaccharides are a kind of plant polysaccharide, which is mostly extracted by water, enzyme and ultrasonic coenzyme methods. The polysaccharide extracted by these methods has the following advantages: (1) the extracted polysaccharide content is high, (2) there are no of organic solvent residues, and (3) it is safer and more reliable in the development of food, drugs and cosmetics [23,24]. The safety and efficacy of *L. japonica* polysaccharides have enabled their use as additives in flower teas, healthy beverages, and throat lozenges. Moreover, they have been used as raw materials for the production of nutritional additives, daily chemical products, cleaning products, and drugs. Overall, the polysaccharide components in *L. japonica* are being tested for use in food, nutrition, medicine, and other fields because of the high development and economic potential.

*L. japonica*, as a common TCM with a long history of use, not only has ornamental value but also has medicinal and nutritional value [25]. The polysaccharides obtained from this plant meet the nutritional requirements of the body. There are literature reviews on *L. japonica*. However, the studies on small-molecule compounds are sorted out, and those on the macromolecular compounds in *L. japonica* polysaccharides are lacking. With the increase of polysaccharide research in recent years, an increasing quantity of *L. japonica* polysaccharides has been extracted, so it has become necessary to summarize and extensively examine research on *L. japonica* polysaccharides. In this review, research on the extraction, purification, chemical structure, health benefits and structure–activity relationship of *L. japonica* polysaccharides over the past 12 years were examined, and the existing applications and potential of *L. japonica* polysaccharides in the food, pharmaceutical, and daily chemical field were summarized. This review will provide knowledge for further research on polysaccharides and other products that include *L. japonica*.

## 2. Extraction and Purification Methods of *L. japonica* Polysaccharides

Polysaccharides are polar macromolecules, which are soluble in water and insoluble in ethanol. Therefore, polysaccharides are extracted using polar solvents, such as water (the principle of similar-phase dissolution) [26]. Traditionally, *L. japonica* polysaccharides are extracted using hot water. Hot water extraction (HWE) is simple to perform, cost-effective, and does not alter the properties of polysaccharides with maximum retention of their activity [27,28,29]. The process is performed multiple times to ensure the complete extraction of the polysaccharides.

The plant is soaked in 95% ethanol for 1–2 weeks to remove the lipophilic components and increase the dissolution rate and polysaccharide yield [30,31]. The yield of *L. japonica* polysaccharides depends on the extraction time and temperature. The extraction time of the HWE was 33–1500 min, the extraction temperature was 45–100 °C, and the total yield was 3.6–7.6%. Several new extraction methods have been developed for *L. japonica* polysaccharides. EAE is a polysaccharide extraction method, which has been extensively used for extracting plant polysaccharides. EAE is an environmentally friendly technology with the advantages of having a simple operation, safety, mild reaction conditions, and being non-toxic [32]. Moreover, the use of EAE improves the extraction efficiency, yield, and biological activity of polysaccharides. During this process, the enzymes break down the plant cell wall into small molecules, then intracellular *L. japonica* polysaccharides are rapidly released in the external medium. However, a combination of multiple methods is preferred to a single extraction method [33,34]. The Ultrasonic-assisted enzymatic extraction (UAEE) method is a combination of ultrasonic extraction technology and enzymatic hydrolysis, and has a high extraction rate, short extraction time, low extraction temperature, and high efficiency [35,36]. UAEE has been used to extract polysaccharides from *L. japonica* leaves. Compared to the HWE process, the UAEE process reduces the extraction time from 8 h to 33 min and increases the yield from 5.3% to 14.76%. Table 1 lists the extraction time, temperature, solid–liquid ratio, total yield, and other characteristics of the different extraction methods of *L. japonica* polysaccharides.

**Figure 2:**
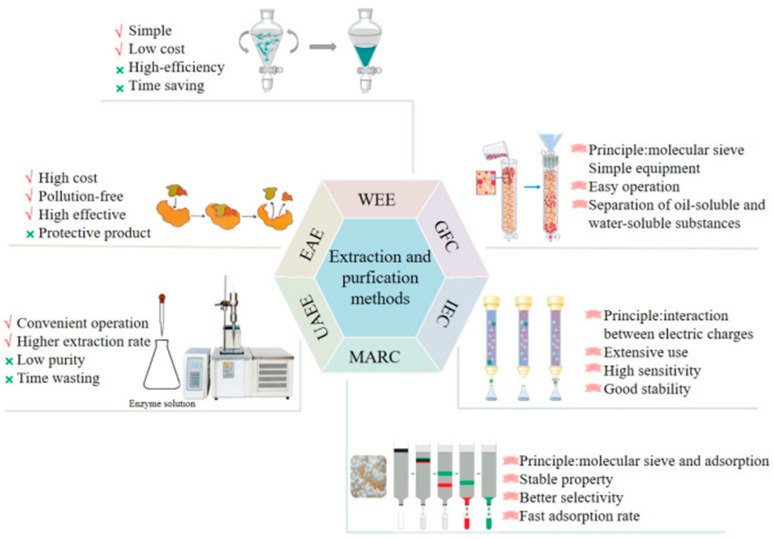
Extraction and purification processes of *L. japonica* polysaccharides.

After the extraction, the crude *L. japonica* polysaccharides are precipitated using ethanol. The extracted polysaccharides are still mixed with impurities, including proteins, pigments and small molecules. The Sevag, trichloroacetic acid, and chloroform-n-butanol methods—based on the principle of protein denaturation in organic solvents—are used for removing these impurities [57,58]. The trichloroacetic acid method is the most frequently used method to remove proteins from *L. japonica* polysaccharide extraction solutions. The solution is neutralized and centrifuged, and the supernatant is collected and dialyzed to remove small-molecule compounds and then dried to obtain the purified extract. A high-purity polysaccharide extract is the primary condition for the experimental analysis of their structure and biological activity. Further, the purified polysaccharide preparations are used to obtain homogeneous polysaccharides. The most common techniques for purifying polysaccharides is by using an ion-exchange column, gel filtration column, or macroporous adsorption resin chromatography [59,60,61]. The schematic representation of the extraction and purification processes of *L. japonica* polysaccharides are shown in Figure 2.

## 3. Structural Characteristics of *L. japonica* Polysaccharides

**Figure 3:**
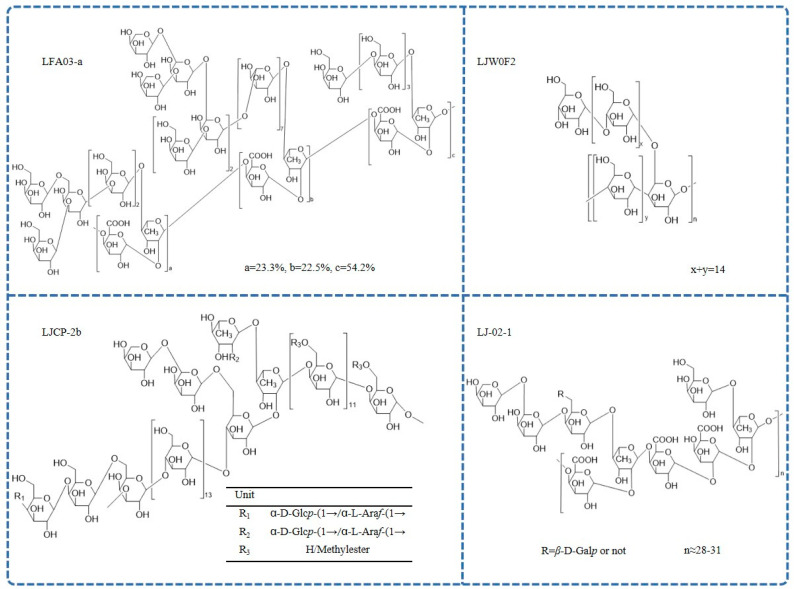
The chemical structure of *L. japonica* polysaccharides.

Different *L. japonica* polysaccharides have different structures and biological properties [62]. Table 2 summarizes the available studies on *L. japonica* polysaccharides, including the name of the compound, molecular weight, monosaccharide composition, structure, and relevant references. The reported chemical structures of polysaccharides are shown in Figure 3.

### 3.1. Molecular Weight

The reported average molecular weights (Mw) of *L. japonica* polysaccharides are variable in different studies because of the variations in plant parts used, extraction methods, purification processes, and analytical methods. For example, the Mw of LJCP-2b extracted from *L. japonica* caulis using HWE was 7.0 kDa, and the Mw of HEP-4 obtained from *L. japonica* flowers using the same extraction method was 198 kDa [49,56]. *L. japonica* flower polysaccharides have an average molecular weight of approximately <1500 kDa. Pectin polysaccharides have a molecular weight range of 7.2–400 kDa, whereas water-soluble polysaccharides have a range of 3.8–198 kDa. A *L. japonica* crude polysaccharide (LJP-b) was completely separated into five purified fractions: LJP-N, LJP-A-1, LJP-A-2, LJP-A-3, and LJP-A-4. Their average molecular weight range identified using linear regression analysis was 3.9–383.8 kDa [39].

### 3.2. Monosaccharide Composition

The content and ratio of monosaccharides provide the basis of the chemical structure of polysaccharides. The study of their structural characteristics is important because the structure is correlated to the biological activity of polysaccharides. With the use of acidic hydrolysis, derivatization, Fourier-transform infrared spectroscopy (FT-IR), nuclear magnetic resonance (NMR) spectroscopy, gas chromatography (GC), and high-performance liquid chromatography (HPLC), the monosaccharide composition of *L. japonica* polysaccharides has been investigated and analyzed [63,64,65,66,67]. The monosaccharide compositions of polysaccharides extracted from the flower, flower bud, stem, branch, and leaf are different, but they are mainly composed of different molar fractions of glucose (Glc), galactose (Gal), rhamnose (Rha), arabinose (Ara), mannose (Man), and xylose (Xyl). Interestingly, ribose (Rib) was detected only in *L. japonica* leaf polysaccharides (LJLP). LJLP was composed of 32.3%, 20.9%, and 15.2% of Gal, Glc, and Rib, respectively [55]. Notably, some *L. japonica* polysaccharides are homogeneous. A monosaccharide composition analysis indicated that LJW0F2 was composed of 99.7% glucose, and LJW2F2 isolated from *L. japonica* flowers was composed of 98.2% galacturonic acid [47,48]. The monosaccharide composition of *L. japonica* polysaccharides extracted from the stem was Glc:GalA:Ara:Gal:Rha:Xyl:Man:GlcA = (3.89:2.49:1.87:1.51:1.00:0.40:0.21:0.19) [56]. In conclusion, the monosaccharide composition of polysaccharide depends partly on the differences in the extraction, purification, and analysis methods.

### 3.3. Chemical Structures

Researchers used infrared spectroscopy (IR), NMR and GC-mass Spectrometry (MS) to characterize the chemical structure of *L. japonica* polysaccharides. We classified the chemical structure of *L. japonica* polysaccharides based on existing research and reports.

Pectin polysaccharides are the most common type of *L. japonica* polysaccharides. A pectic polysaccharide named LFA03-a was obtained from the water extract of *L. japonica* flowers for the first time. The structure of LFA03-a included a rhamnogalacturonan I (RG-I) backbone consisting of repeating units of *α*-l-1,2-Rha*p* and *α*-d-1,4-GalA*p* disaccharide with a substitution of rhamnose at C4. The side chain was involved with *β*-d-1,4-Gal*p*; *β*-d-1,3-Gal*p*; *β*-d-1,3,6-Gal*p*; and branched *α*-l-1,5-Ara*f* [21]. Similarly, the structure of another pectin polysaccharide, WLJPA0.2b, was studied using FT-IR spectra, enzymatic hydrolysis, de-esterification, partial acid hydrolysis, NMR spectra, and electrospray ionization-mass spectrometry (ESI-MS) analysis. This polysaccharide consisted of homogalacturonan (HG) along with RG-I and RG-II domains (mass ratio 2.1:0.4:1.0). Highly branched *α*-l-1,5-arabinan; *β*-d-1,4-galactan; and type II arabinogalactan side chains were found in the RG-I domain [44]. In addition, another pectin polysaccharide, LJ-02-1, was also isolated, and its backbones comprised→4)-*α*-d-Gal*p*A-(1→2)-*α*-l-Rha*p*-(1→, which was substituted partly at the C4 position of rhamnose by side chains, including 1,4,6-linked *β*-d-Gal*p*; 1,5-linked *α*-l-Ara*f*; T-linked *α*-l-Ara*f*; and T-linked *β*-d-Gal*p* [46].

Further, a limited number of reports on the structure of neutral *L. japonica* polysaccharides are also available. Methylation, HPLC, FT-IR, and extensive 1D- and 2D-NMR were used to examine the detailed structure of a neutral polysaccharide (LJW0F2) from *L. japonica* flowers. The absorption at 929.2 cm^−1^ in the infrared spectrum is a typical signal of d-Glc in pyranose. In the ^13^C NMR spectrum, only one anomeric resonance signal at δ101.00 was present, indicating *α*-glycosidic linkages. The result showed that the linkages of LJW0F2 consisted of terminal glucose; 1,4-linked glucose; and 1,4,6-linked glucose in the molar ratio of 1:14:1 [47]. In addition, there was a neutral-fraction LJP-N that was a starch-like glucan with some arabinogalactan or arabinan domains [37]. Another homogeneous heteropolysaccharide, LJCP-2b, mainly consisted of 1,3,6-*β*-d-Gal*p*; 1,4-*α*-d-Glc*p*; 1,4,6-*α*-d-Glc*p*; 1,4-*β*-d-Gal*p*; 1,2,4-*α*-l-Rha*p*; and 1,4-*α*-d-Gal*p*A [56]. FT-IR spectrometric analysis showed that LJP-d had typical polysaccharide absorption characteristics at 4000–100 cm^−1^, which was caused by the vibration of O-H bonds and C-H bonds in sugar compounds and the crystalline water of sugar compounds [42].

## 4. Health Benefits of *L. japonica* Polysaccharides

**Figure 4:**
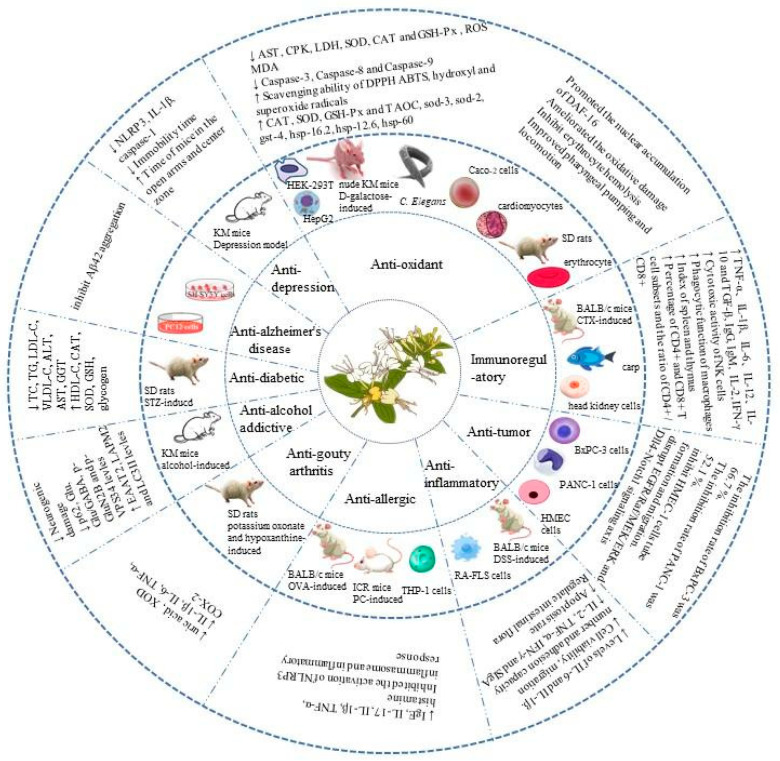
Health benefits of *L. japonica* polysaccharides.

*L. japonica* is a medicinal and edible plant with great research and development value [68]. As one of its active ingredients, *L. japonica* polysaccharides have anti-diabetic, anti-Alzheimer’s disease, anti-depression, antioxidation, immunoregulatory, anti-tumor, anti-inflammatory, anti-allergic, anti-gout, and anti-alcohol-addiction activities. Many in vitro and in vivo studies have been conducted on the biological activity and health benefits of *L. japonica* polysaccharides. Table 3 presents comprehensive information on the health benefits of *L. japonica* polysaccharides. The combined health benefits are shown in Figure 4.

### 4.1. Anti-Diabetic Effects

Diabetes mellitus is a global public health problem and is a group of metabolic diseases caused by insulin secretion and utilization disorders, which have multiple causes [69]. LJP-w, a crude polysaccharide purified from the water extract of *L. japonica*, had hypoglycemic and hypolipidemic effects in a rat model of streptozotocin (STZ)-induced diabetes, and it protected the mice from STZ-induced oxidative stress. Compared with the levels in the model group, the oral administration of 200, 400, and 800 mg/kg LJP-w for six weeks markedly reduced fasting blood glucose levels in the treatment groups with a notable reduction in body weight, food consumption, and water intake. In addition, the pyruvate kinase concentrations and hexokinase activity in diabetic rats treated with LJP-w were restored from 37.6 ± 5.8 and 5.0 ± 0.8 U/g protein to 107.70 ± 6.90 and 8.80 ± 0.70 U/g protein, respectively, suggesting that LJP-w alleviated insulin resistance. The levels of total cholesterol (TC), triglyceride (TG), low-density lipoprotein-cholesterin (LDL-C), very-low-density lipoprotein-cholesterin (VLDL-C), and high-density lipoprotein-cholesterin (HDL-C) in serum were detected using commercial enzyme kits. The results indicated that treatment with 800 mg/kg LJP-w significantly decreased the levels of plasma TG, TC, LDL-C, and VLDL-C to 1.60 ± 0.20, 1.70 ± 0.20, 0.47 ± 0.07, and 1.03 ± 0.32 mmol/L, respectively, and significantly increased the HDL-C levels to 0.79 ± 0.07 mmol/L (*p* < 0.05). These values were similar to those obtained in the normal control group. These findings indicated that LJP-w had an inhibitory effect on the lipid content in the blood. Moreover, LJP-w increased the antioxidant capacity of serum and liver. Oxidative stress is a core pathogenic mechanism in diabetes. The levels of alanine transaminase (ALT), aspartate transaminase (AST), and gamma-glutamyl transpeptidase (GCT) in the sera of diabetic rats treated with 800 mg/kg LJP-w decreased to 48.70 ± 7.7, 81.90 ± 15.10, and 38.30 ± 4.60 IU/L, respectively, whereas the levels of catalase (CAT), superoxide dismutase (SOD), and glutathione (GSH) in the liver increased to 84.50 ± 8.20 U/mg, 300.90 ± 23.00 U/mg, and 9.80 ± 0.70 mg/g protein, respectively. Further, the effects of LJP-w on the activities of *α*-amylases and *α*-glucosidases were evaluated in vitro using acarbose as a positive control. These two enzymes hydrolyze polysaccharides, and the inhibition of their activity decreases the production of glucose, thereby controlling postprandial blood sugar levels. The IC_50_ values of LJP-w for *α*-amylase and *α*-glucosidase were 61.2 ± 3.1 and 45.6 ± 1.9 μg/mL, respectively, which were comparable with those of the acarbose treatment group. Taken together, LJP-w exerts anti-diabetic effects by reducing hyperglycemia, decreasing hyperlipidemia, and increasing the antioxidant capacity of serum and liver. Therefore, LJP-w can be incorporated into functional foods for reducing blood glucose levels and may be used for designing novel therapeutic strategies for diabetes [53].

### 4.2. Anti-Alzheimer’s Effects

Alzheimer’s disease (AD) is the most common type of senile dementia, which threatens the physical and mental health of the elderly population worldwide. “Amyloid hypothesis” is currently recognized as the main pathogenic mechanism of Alzheimer’s disease. The accumulation of misfolded A*β* peptides in the central nervous system results in plaque formations in the brain and the disruption of neuronal function, and these A*β* aggregates have been extensively studied to develop treatment strategies for AD [70,71]. Current research focuses on blocking A*β* aggregation and reducing its neurotoxicity for the prevention and treatment of AD [72,73]. Thioflavin T (ThT) as well as atomic force microscopy (AFM) and fluorescence spectroscopy were used to determine the inhibitory effect of different doses of a glucan (JLW0F2), which was isolated from *L. japonica,* on A*β*_42_ aggregation. Compared with that of A*β*_42_ incubated alone, the aggregation inhibition rate of A*β*_42_ incubated with 200 g/mL of LJW0F2 was >90% in the ThT spectroscopic assay. The results of the AFM suggested that LJW0F2 delayed the formation of A*β*_42_ fibrils in a dose-dependent manner. Further, the neurotoxicity of A*β*_42_ was evaluated by incubating SH-SY5Y cells (human neuroblastoma cells) with A*β*_42_ alone and with different concentrations of LJW0F2 (0.1, 1, and 10 g/mL) for seven days, and the viable cell count was determined using the CCK-8 assay. Compared to the control treatment, the LJW0F2 treatment attenuated the cytotoxicity of the A*β*_42_ aggregates on the SH-SY5Y cells [47]. Subsequently, the research group determined that a pectin polysaccharide extracted from *L. japonica* (LFA03-a) also inhibited A*β*_42_ aggregation. In addition, LFA03-a induced the differentiation of PC12 neuronal cells derived from a transplantable rat pheochromocytoma to promote neuritogenesis. The PC12 cell line was used to determine the degree of neuronal differentiation and neurosecretion after the LFA03-a treatment to investigate the potential effect of LFA03-a on neurite outgrowth, and 25 ng/mL of nerve growth factor (NGF) was used as a positive control. The results showed that the LFA03-a (1 mg/mL) treatment promoted the neurogenesis of PC12 cells [21]. In summary, the polysaccharides LJW0F2 and LFA03-a can inhibit the aggregation of A*β*_42_, reduce neurotoxicity, and promote neurogenesis. These results suggest that *L. japonica* polysaccharides may be used for the prevention and treatment of neurodegenerative disorders and nervous system injuries. However, further research is required in this context.

### 4.3. Anti-Depressant Effects

Depression is a common disease worldwide with increasing morbidity and mortality, and its main clinical features include low mood, slowed thinking, decreased will, despair for the future, and even suicidal thoughts [74]. LJP-l, a polysaccharide from *L. japonica*, has anti-depressant effects. Seven different methods were used to randomly stimulate mice for 21 days for establishing a depression model. The behavioral tests of mice, including open-field, elevated plus maze, tail suspension, and forced swim tests, were performed after administrating fluoxetine (Flu) and LJP-l. The LJP-l treatment increased the time of opening arms and reduced the resting time of depressed mice. The LJP-l treatment also increased the number of nerve cells and ameliorated the irregular arrangement and deformation of nerve cells in the hippocampus of depressed mice. Western blot analysis showed that LJP-l protected the nerve cells in depressed mice by inhibiting the NLRP3 inflammasome pathway and reducing the production of caspase-1 and IL-1*β*. However, the effect of LJP-l was more significant than Flu, suggesting that the NLRP3 inflammasome pathway may not be targeted by drugs. Therefore, the NLRP3 inflammasome may be involved in the pathogenesis of depression [52]. However, more animal experiments and clinical studies are needed to further confirm the effect of *L. japonica* polysaccharides for treating depression.

### 4.4. Antioxidant Effects

Oxidative stress is a state of imbalance of free radicals and antioxidants in the body, which results in molecular and cellular damage. Antioxidants protect the body from free radical damage by scavenging the free radicals [75]. A water-soluble polysaccharide, HEP-4, extracted from *L. japonica* exerted antioxidant effects on human hepatoma cells (HepG2). The antioxidant activity of HEP-4 was evaluated using four free-radical scavenging methods, namely 1,1-diphenyl-2-picryl-hydrozyl, (DPPH); hydroxyl (OH); 2,2-azidobisphenol (3-ethylbenzothiazoline-6-sulfonic acid) (ABTS); and superoxide radical scavenging tests. The results showed that the scavenging activities of the DPPH, OH, ABTS, and superoxide radical increased with the increase in the HEP-4 concentration, and the scavenging rates were 69.7%, 40.6%, 92.5%, and 39.8%, respectively, at 1.0 mg/mL of HEP-4. In addition, 800 μg/mL of HEP-4 reduced the concentrations of reactive oxygen species (ROS) and malondialdehyde (MDA) as well as enhanced the activity of CAT and glutathione peroxidase (GSH-Px). All in all, HEP-4 had a protective effect on H_2_O_2_-exposed HepG2 cells [49]. WLJP-A0.2b, a pectin polysaccharide, was derived from *L. japonica*, and it was composed of RG-I (WLJP-A0.2b-E1), RG-II (WLJP-A0.2b-E2), and HG domains. Enzymatic hydrolysis of the HG domain produced unesterified and partly methyl-esterified and acetyl-esterified oligogalacturonides (WLJP-A0.2b-E3). The antioxidant activity of different domains was compared, and the results of the DPPH radical scavenging test indicated that the IC_50_ values for WLJP-A0.2b-E1, -E2, and -E3 were 125.27 ± 0.91, 86.81 ± 0.41, and 27.06 ± 0.53, respectively. The evaluation of HEK-293T cells treated with H_2_O_2_ showed that oligogalacturonides protected HEK-293T cells from oxidative damage by inhibiting ROS production [44]. Four acidic fractions (LJP-A-1 to LJP-A-4) and a neutral fraction (LJP-N) were obtained from the crude polysaccharide fraction of *L. japonica*, and their antioxidant potential was determined using six methods [39]. In addition, LJLP (heteropolysaccharide) showed antioxidant activity in a mouse model of d-galactose-induced oxidative stress. LJLP decreased the MDA concentrations, increased the activities of CAT, SOD, and GSH-Px, and improved the total antioxidant capacity (TAOC) in the serum and liver of mice. Simultaneously, the scavenging rate of LJLP for superoxide radicals was detected in vitro with ascorbic acid as the standard. The results indicated that the concentration of LJLP was positively correlated with its clearance rate, and LJLP showed a stronger clearance ability when at concentration of 1.0 mg/mL [55].

Oxidative stress is one of the important factors leading to aging and disease. Aging is inevitable; however, it is possible to delay aging [76,77]. *Caenorhabditis elegans* (*C. elegans*) and Caco-2 cells were used as models to study the anti-aging and antioxidant effects of crude *L. japonica* polysaccharide (CLJP) and its purified fraction (LJP-2-1). The anti-aging activity was evaluated by observing the lifespan, exercise capacity, lipofuscin accumulation, and the regulation of daf-16, sod-3, gst-4, and hsp genes of *C. elegans* before and after administrating the polysaccharides. The results showed that the average lifespans of nematodes in the groups treated with 200 μg/mL of CLJP and LJP-2-1 were prolonged by 13.97% and 11.35%, respectively, and the lipofuscin concentration was lower in the treated groups compared to that in the control group. CLJP did not have any negative effect on the fecundity and body length of nematodes and had a protective effect on oxidative and heat stress in nematodes. The CLJP treatment had an antioxidant effect and increased the activity of antioxidant enzymes in *C. elegans*. Compared with that of the control group, the SOD activity of the CLJP (400 μg/mL) group increased by 2.81 ± 0.05-fold (*p* < 0.001), and the CAT activity increased by 2.32 ± 0.07-fold (*p* < 0.001). The 3-(4,5-Dimethylthiazol-2-yl)-2,5-diphenyl terazolium bromide (MTT) assay showed that the CLJP pretreatment notably alleviated the H_2_O_2_-induced oxidative damage in Caco-2 cells. In addition, the nuclear localization of HSP-16.2⸬GFP in the CLJP (200 μg/mL) group was 28.97% higher than that in the control group (*p* < 0.01), and the mRNA expression levels of daf-16, dod-3, skn-1, and daf-12 were significantly higher than those in the control group. Therefore, CLJP affected the lifespan and health of nematodes by mediating DAF-16 [45]. An excess of free radicals in the human body can cause atherosclerosis, neurologic diseases, and other diseases. Therefore, antioxidants are crucial in the prevention and management of cardiovascular abnormalities. The *L. japonica* polysaccharide LJP-z had a protective effect on cardiomyocytes injured by H_2_O_2._ The LJP-z-treatment (10, 20, and 40 μg/mL) enhanced the viability of injured cardiomyocytes and reduced cell damage and apoptosis [41]. Moreover, a rat model of ischemia/reperfusion was established using the modified middle cerebral artery occlusion (MCAO) method to study the neuroprotective effect of a water-soluble polysaccharide (LJPB2). The polysaccharide reduced the MDA concentration, decreased NO production and enhanced the activity of SOD and GSH-Px. Further, it showed strong DPPH free-radical scavenging ability in vitro [22]. Taken together, *L. japonica* polysaccharides have excellent antioxidant activity and may be used as effective therapeutic agents for oxidative-stress-related diseases.

### 4.5. Immunoregulatory Effects

Immune regulation is the most important biological activity of polysaccharides. Immunologically active polysaccharides have been extensively studied because of their numerous sources, low price, positive effects, purity, and minimal side effects [78]. Several studies have reported the immunomodulatory activity of *L. japonica* polysaccharides and have compared the immunostimulatory activities of the two *L. japonica* polysaccharides, LJP-N and LJP-A. A single intraperitoneal injection of cyclophosphamide (CTX) induced immunosuppression in BALB/c mice, then LJP-N (50 mg/kg) and LJP-A (200 mg/kg) were intragastrically administered to these mice. Compared with those in CTX model mice, the spleen and thymus indexes were higher in mice treated with LJP-N and LJP-A, and the spleen index of the LJP-A group was significantly higher than that of the LJP-N group. In addition, the concentrations of interleukin (IL)-2, IL-6, tumor necrosis factor (TNF)-α, immunoglobulin (Ig)G, and IgM in the LJP-A-treatment group were increased by approximately 2.0-, 2.5-, 1.4-, 1.7-, and 1.2-fold compared with those in the CTX model group (*p* < 0.001), suggesting that LJP-A exerted an immunomodulatory effect by increasing the cytokine secretion and immunoglobulin concentrations. Simultaneously, the phagocytosis of the LJP-N and LJP-A components were determined by injecting India ink into the tail vein of the female BALB/c mice. The phagocytic rate and index in the LJP-A groups were markedly higher at both low and high concentrations (50 and 200 mg/kg, respectively) of polysaccharide, whereas the phagocytosis parameters in the LJP-N group were significantly higher only at a high concentration (200 mg/kg). In addition, both LJP-N and LJP-A enhanced the cytotoxic activity of natural killer (NK) cells. However, the apoptosis rate of YAC-1 cells (the targets of NK cells) in the LJP-A-treated mice was higher than that in the LJP-N-treated mice [37]. In a similar study, LJP-d, a polysaccharide extracted from *L. japonica* using EAE, improved the immune function in CTX-induced immunosuppressed mice. Further, LJP-d increased the CD4+/CD8+ T-cell counts, thereby suggesting that it enhanced the cellular immune response in the immunosuppressed mice [42]. The immunomodulatory activity of a water-soluble polysaccharide (HP-02) from *L. japonica* was evaluated in carp fish. The researchers evaluated the effects of different concentrations of HP-02 (250, 500, and 1000 μg/mL) on the proliferation and phagocytic activity of head kidney cells and the secretion of cytokines in cephalic kidney cells extracted from the common carp. The results showed that the HP-02 treatment increased proliferative and phagocytic activity and stimulated the secretion of TNF-α, IL-1*β*, IL-6, IL-10, and transforming growth factor-*β* (TGF-*β*) in the head kidney cells of carp fish. In addition, the serum concentrations of these cytokines were higher in the HP-02-treated fish than in the untreated fish. Thus, HP-02 showed notable immunoenhancement effects in carp fish [51]. Overall, *L. japonica* polysaccharides can be potentially used as adjuvant immunomodulators in therapeutic drugs and functional foods.

### 4.6. Anti-Tumor Effects

Tumors are solid tissue masses formed by abnormal cells, which are categorized as benign and malignant tumors (cancers). Cancer has become one of the most serious diseases endangering human health, and its incidence and mortality are increasing worldwide [79]. Pancreatic cancer is one of the common malignant tumors of the digestive tract (also known as the “king of cancer”). The 5-year survival rate of pancreatic cancer after diagnosis is only 10% with negligible treatment options [80]. A homogeneous polysaccharide, LJ-02-1, from the crude polysaccharide of *L. japonica* was evaluated for anti-pancreatic cancer effects. BxPC-3 and PANC-1 pancreatic cancer cells were treated with different concentrations of LJ-02-1 (0.016, 0.031, 0.063, 0.125, 0.250, 0.500, and 1 mg/mL) for 72 h. The results of the MTT assay showed that the inhibition rates of the proliferation of BxPC-3 and PANC-1 cells treated with 1 mg/mL LJ-02-1 were 66.7% and 52.1%, respectively, in a dose-dependent manner. Moreover, LJ-02-1 was minimally cytotoxic to normal liver cells (LO2) with only a 26.2% inhibition rate [46]. Anti-angiogenesis therapy is a new strategy for tumor treatment in recent years and has shown remarkable therapeutic outcomes in several cancers [81,82,83]. A homogeneous polysaccharide, LJW2F2, blocked angiogenesis by affecting the formation of capillary-like tubes and cell migration in HMEC-1 (human microvascular endothelial cells). Epidermal growth factor receptor (EGFR), which is essential for cell proliferation and signal transduction, is converted from monomer to dimer after binding with specific ligands. The autophosphorylation of this dimer guides the phosphorylation of the downstream Raf/MEK/ERK pathway in angiogenesis. LJW2F2 inhibits the phosphorylation of EGFR and its downstream pathways. In addition, LJW2F2 also partially inhibits the Notch1/Dll4 signaling pathway. Therefore, the anti-tumor mechanism of LJW2F2 is associated with the inhibition of the EGFR/Raf/MEK/ERK and Dll4/Notch1 signaling pathways [48]. Therefore, *L. japonica* polysaccharides have potential applications in anti-tumor therapies.

### 4.7. Anti-Inflammatory Effects

Inflammation is a defense mechanism activated in response to tissue injury. Immune-mediated inflammatory disease (IMID) is a highly disabling chronic disease that affects different organs and systems and deteriorates the health and quality of life of patients [84]. Ulcerative colitis (UC) and rheumatoid arthritis (RA) are the two common chronic inflammatory diseases. UC is a refractory chronic intestinal disease with a complex etiology, and the inflammatory process caused by the imbalance of the intestinal mucosal immune system participates in the pathogenesis of UC [85]. The anti-UC activity of the *L. japonica* polysaccharide LJP-k was evaluated in a DSS-induced UC mouse model. Changes in body weight, spleen, and thymus indexes reflect the innate immune function of the body. The body weight, spleen, and thymus indexes decreased in the UC mice; however, the values were markedly restored after the LJP-k treatment. The LJP-k (50, 100, and 150 mg/kg) treatment increased the concentrations of IL-2, TNF-*α*, interferon (IFN)-*γ*, and secretory immunoglobulin A (SIgA) in UC mice in a dose-dependent manner, indicating that LJP-k regulated their immune imbalance. Moreover, LJP-k regulated intestinal disorders and maintained intestinal microbiota homeostasis. Compared with those in the UC mice, the colony-forming units (CFUs) of *Bifidobacterium* and *Lactobacillus* were gradually restored, whereas the CFUs of *Escherichia coli* and *Enterococcus* were reduced in the cecum of LJP-k-treated mice. The flow cytometry results indicated that the apoptosis rate of splenic lymphocytes in UC mice gradually recovered after the intragastric administration of LJP-k. Therefore, *L. japonica* polysaccharides are potential anti-UC molecules [40].

RA is a chronic inflammation characterized by erosive arthritis [86]. Fibroblast-like synoviocytes (FLSs) are the main cellular components in the inflamed joints of patients with RA. The anti-RA effect of LJCP-2b, a homogeneous heteropolysaccharide from *L. japonica* caulis was evaluated in FLS cells treated with 20 ng/mL TNF-*α*. The results showed that LJCP-2b inhibited cell viability as well as IL-6 and IL-1*β* secretion, and promoted the apoptosis of RA-FLS. The effect of LJCP-2b on the migration of RA-FLS cells was measured using the transwell assay. The results showed that LJCP-2b (50–200 μg/mL) significantly reduced the migration and adhesion of RA-FLS (*p* < 0.01), thereby alleviating the increase in inflammation [56]. Altogether, *L. japonica* polysaccharides can be developed as potential anti-inflammatory drugs in the future.

### 4.8. Anti-Allergic Effects

An allergy is when an allergen binds to an antibody to produce a series of allergic reactions in the body [87]. *L. japonica* polysaccharides have therapeutic effects on allergic contact dermatitis (ACD) and allergic rhinitis (AR). A picryl chloride (PC)-induced ACD mouse model was used to evaluate the anti-allergic effect of a water-soluble polysaccharide, LJP-1-t, with prednisolone as a positive control. LJP-1-t was orally administered at 15 h before and after PC stimulation. The LJP-1-t treatment significantly reduced the ear swelling rate, whereas the weight of the thymus, spleen, and adrenal gland were unchanged. PC induction significantly increased the serum concentrations of IgE and TNF-*α*. The LJP-1-t treatment (20, 40, and 80 mg/kg) decreased IgE and TNF-*α* concentrations, and the therapeutic effect of a high-dose of LJP-1-t was similar to that of prednisolone. Interestingly, LJP-l-t also inhibited the increase in serum histamine concentrations [54]. AR affects a large proportion of the world population and is a major socioeconomic burden [88]. A pectin polysaccharide, WLJP-025p, alleviated ovalbumin (OVA)-induced AR in a mouse model. The mechanism of action involved inhibiting the activation of the NLRP3 inflammasome, regulating the immune system, and maintaining the balance of intestinal microbiota. After an intraperitoneal injection of 30 and 60 mg/kg WLJP-025p, the concentrations of IgE, IL-17, and IL-1*β* were significantly decreased, and the behavioral symptoms of sneezing and rubbing were markedly ameliorated in the AR mice. In addition, phorbol 12-myristate 13-acetate (PMA)-induced THP-1 (a human acute monocytic leukemia cell line) cells were incubated with lipopolysaccharide (LPS) and IFN-*γ* to establish an AR model for evaluating the effect of WLJP-025p. The WLJP-025p treatment inhibited the activation of the NLRP3 inflammasome and inflammatory response (an NLRP3 inhibitor, CY-09, was used as a positive control). Therefore, NLRP3 may be the possible target of WLJP-025p to alleviate AR [50]. The anti-allergic effect of *L. japonica* polysaccharides can be further explored for developing novel therapeutic approaches for allergic diseases.

### 4.9. Anti-Gout Effects

Gout is a disease caused by the deposition of monosodium urate crystals (MSU) in the joints because of high blood uric acid levels. The deposited crystals cause painful inflammation in and around the joints. Hyperuricemia is the pathologic basis of gout. The clinical detection of hyperuricemia has increased over the years. Hyperuricemia is the ‘fourth highest’ lifestyle disease after hypertension, diabetes, and hyperlipidemia, and the younger population is increasingly being affected by this disease [89]. LJP-1-y, a polysaccharide extracted from *L. japonica*, was shown to have anti-hyperuricemia properties. Hyperuricemia was induced in SD rats using an intraperitoneal injection of a potassium oxide emulsion and the intragastric administration of a hypoxanthine suspension. LJP-1-y (100, 200, and 300 mg/kg) was given to the rats 1 h after establishing the model, and the positive control group was treated with allopurinol (10 mg/kg of body weight). The LJP-1-y treatment reduced serum uric acid concentration and inhibited xanthine oxidase (XOD) activity in a dose-dependent manner. Notably, a sustained increase in serum uric acid levels and the accompanying deposition of MSU in the joint membranes, synovium, cartilage, bones, and other joint tissues leads to gouty arthritis. In a rat gouty arthritis model established by injecting MSU crystals, LJP-1 ameliorated the degree of ankle swelling in rats and decreased the IL-1*β*, IL-6, TNF-*α*, and cyclooxygenase-2 (COX-2) levels in the serum, eventually alleviating gouty arthritis [43]. Compared to commonly used anti-gout drugs, such as nonsteroidal anti-inflammatory drugs (NSAIDs), colchicine, glucocorticoids, and allopurinol, *L. japonica* polysaccharides are safe and effective for the treatment of gout with minimal side effects. Therefore, LJP-l can be further developed as an anti-gout drug.

### 4.10. Anti-Alcohol-AddictionEffects

Problematic drinking behavior is a public health concern worldwide and leads to serious physical, psychological, and social damage. *L. japonica* polysaccharides can treat alcohol addiction and slow down the physical damage caused by excessive alcohol consumption [90,91]. A *L. japonica* polysaccharide, LJP-s, was obtained using HWE and its effect on conditioned place preference (CPP) and alcohol addiction memory were investigated in an alcohol-induced CPP mouse model. The expression of glutamate (Glu), *γ*-aminobutyric acid (GABA), and Glu transporters as well as receptors in the hippocampus of the LJP-treated CPP mouse model was evaluated. The results suggested that LJP-s ameliorated alcohol-induced CPP in mice by regulating the expression of EAAT2 and the phosphorylation of GluN2B and inhibiting the increase in the Glu concentration and Glu/GABA ratio. The effects of LJP-s on addiction and memory were further analyzed using Western blotting. The results showed that the LJP-s treatment decreased the concentrations of the autophagy regulators p-VPS34, ULK, and p-beclin-1 and increased the concentrations of the autophagy proteins p-62, LAPM2, and LC3II. In addition, the VPS34 inhibitor (VPS34-IN-2) had a suppressive effect on the alcohol-induced reinstatement of CPP. Taken together, LJP-s inhibited the activation of autophagy in the hippocampus by mediating VPS34 phosphorylation, thereby reducing the occurrence of alcohol-related diseases. Moreover, the Nissl assay and immunofluorescence staining results indicated that LJP-s alleviated nerve injury and pathologic changes [38]. Therefore, LJP-s could be used as an anti-alcohol-addiction agent and in new functional foods.

## 5. Structure–Activity Relationship and Structural Modifications

The chemical structure of a polysaccharide is the material basis for its biological activity, and its complexity determines various pharmacologic effects [92]. The molecular weight, monosaccharide composition, and molecular main chain and spatial conformation of polysaccharides have notable effects on their biological activities. LJP-N (MW: 5.4 kDa) is composed of Glc:Gal:Ara, and LJP-A (MW: 400 kDa) is composed of GalA:Gal:Ara. The phagocytic rate and index of LJP-A-treated (50 mg/kg) phagocytic cells were significantly higher than those of LJP-N-treat (50 mg/kg) cells. The higher immunomodulatory activity of LJP-A may be attributed to the presence of more GalA residues and greater molecular weight. Interestingly, the biological activities varied with the different proportions of the same monosaccharide composition. LJP-l and HEP-4 were composed of GalA:Rha:Gal:Ara:Glc:Man in the ratios of 8.7:8.2:16.2:19.5:26.9:20.5 and 1.04:1.56:4.31:5.4:14.21:6.74, respectively. LJP-l had notable anti-depressant properties, and HEP-4 reduced oxidative stress. Similarly, WLJP-A0.2b and LJCP-2b were composed of different proportions of Glc:GalA:Ara:Gal:Rha:Xyl:Man:GlcA and showed anti-inflammatory and antioxidant effects, respectively. Moreover, biological activity is also related to different domains of a polysaccharide. WLJPA0.2b consists of RG-I, RG-II, and HG domains. The antioxidant capacity of oligogalacturonates (specifically the de-esterified ones) produced by degrading the HG domain was higher than that of the RG-II and RG-I domains. The degree of methyl esterification, galacturonic acid concentration, and molecular weights of various domains all have an impact on biological activity [9], Notably, the main chain of polysaccharide molecules has a major contribution to its biological characteristics. The main chain of LFA03-a and LJ-02-1 is composed of a repeating unit: →4)-*α*-d-Gal*p*A-1-(1→2)-*α*-l-Rha*p*-(1→. However, the two polysaccharides show different pharmacologic activities, i.e., anti-Alzheimer’s disease and anti-tumor activities. This may occur because LFA03-a has more side chains of *β*-D-1,4-Gal*p*; *β*-d-1.3-Gal*p*; and *β*-d-1,3,6-Gal*p* than LJ-02-1. In contrast, LJ-02-1 has more T-1,4,6-linked-*β*-d-Gal*p* side chains than LFA03-a. The advanced structure of polysaccharides often has a greater impact on its activity than the primary structure. A Congo red experiment confirmed that LJP-1-y has a triple helix conformation. This may indirectly affect its effect on the treatment of gout. However, the connection between the spatial conformation and biological activity of polysaccharides remains to be further studied.

Structural modifications are important means to enhance the biological activity of polysaccharides [93]. The modifications are mainly achieved using chemical, physical, and biological methods [94,95]. Among them, chemical modification is the most widely used method for incorporating structural modifications in polysaccharides. However, a limited number of studies have reported the structural modification of *L. japonica* polysaccharides. Selenium (Se) polysaccharide was formed by an organic combination of Se and the polysaccharides of *L. japonica*. Compared to *L. japonica* polysaccharides, *L. japonica* Se polysaccharides markedly enhanced the proliferation of mouse monocyte/macrophage-like RAW268.7 cells and promoted the production of IL-1*β* and TNF-*α*. The structure–activity relationship of *L. japonica* polysaccharides provides a reference for the directional synthesis and design of polysaccharide drugs and lead compounds.

## 6. Practical and Potential Applications of *L. japonica* Polysaccharides

### 6.1. In the Food Industry

*L. japonica* can be incorporated into-the-one’s-daily-diet as a health supplement or functional food. Its polysaccharide components have numerous biological activities. *L. japonica* flower tea has several beneficial effects on health, such as reducing blood pressure, lowering serum cholesterol, lowering blood sugar, and preventing cardiovascular diseases [96]. *L. japonica* polysaccharides dissolve in water and improve the tolerance of the body to hypoxic free radicals, enhance memory, and delay aging. Therefore, these polysaccharides can be used to make energy drinks or can be added to commercially available drinks. A *L. japonica* functional yogurt has been developed using *L. japonica* and fresh milk. It is a new health drink that integrates the health benefits of *L. japonica* and yogurt. After an analysis of *L. japonica* polysaccharides, they were shown to played a role as a stabilizer in the product and were further refined and developed into a yogurt-additive stabilizer for a variety of dairy products. In addition, *L. japonica* polysaccharides can be used as additives in pasta and noodles for increasing their nutritional value and health benefits. Further, *L. japonica* polysaccharide has been used as a sugar group to improve the efficiency of fermentation and the quality of wine. A distiller’s grains containing *L. japonica* polysaccharide can be brewed into soy sauce with improved aroma and flavor and high nutritional value. Therefore, *L. japonica* polysaccharides have potential applications in the fermentation industry. Moreover, they can be used to develop a range of products, such as jellies and candies. The addition of *L. japonica* polysaccharides in fruit juices containing pulp can reduce the formation of precipitates that are difficult to disperse. These polysaccharides can be added to diet soft drinks to enhance their taste and nutritional value. Currently, several food products containing *L. japonica* polysaccharides (including flower tea, yogurt, noodles, and jellies) and others fermented with *L. japonica* (wine, soy sauce, and vinegar) are available in the market.

### 6.2. In the Pharmaceutical Industry

*L. japonica* is used as a medicinal plant in TCM because of its pharmacologic activities. *L. japonica* crude extracts or their active components are used as raw materials for therapeutic drugs or as medicinal extracts. The small-molecule components include essential oils, saponins, organic acids, iridoids, and flavonoids, and the macromolecules include polysaccharides, which are present in a large quantity with potent biological activities [97]. Although the small-molecule components of *L. japonica* have been more extensively studied, macromolecular polysaccharides have also shown good application potential. Notably, the extraction of polysaccharides from the portions obtained after extracting small-molecule components will greatly increase the economic value of *L. japonica*. For example, acidic pectin polysaccharides (20–400 kDa), which have good emulsification, gelation, and stability features, have been extensively studied and used for commercial purposes. Several *L. japonica* polysaccharides have pharmacologic activities, which explains the therapeutic effect of *L. japonica* in multiple diseases. Therefore, these polysaccharides have the potential of being developed into new drugs. Lianhua Qingwen, a Chinese patent medicine containing *L. japonica*, has good antiviral, immunomodulatory, and anti-inflammatory activities. It has been prescribed for the prevention and treatment of coronavirus infections [98]. According to the performance report of Lianhua Qingwen’s manufacturer Yiling Pharmaceutical, in 2022, it is expected to achieve net profit of CNY 21.5 to 24.19 billion, an increase of 60% to 80% over the same period last year, and its highest net profit has been close to the sum of 2020 and 2021. The active compounds (including polysaccharides) of *L. japonica* are known to have antiviral properties, and recent studies have suggested that the *L. japonica* polysaccharides have immune regulatory and anti-inflammatory effects. Shuanghuanglian, made from *L. japonica* as the core TCM, is commonly used to treat inflammation in China. Its frequency of use has been comparable to amoxicillin [99]. The anti-inflammatory and immunomodulatory effects of polysaccharides have been extensively studied by pharmaceutical companies. An antiviral polysaccharide compound tablet was invented, made of *L. japonica* polysaccharides and other 11 kinds of polysaccharide in accordance with the ratio of 22:6:7:10:4:7:9:6:7:13:4:2:3 mixed evenly, and it is recommended that it be taken three times a day, one piece each time, for the treatment of influenza. It has the advantages of being convenient to take and has shown a definite curative effect, i.e., greatly reducing pain in patients. *L. japonica* polysaccharides and other polysaccharide components play a synergistic role in preventing and treating tumors through antibacterial and immune-enhancing effects, thus realizing the possibility of polysaccharide components as patented medicine. In addition, lozenges containing *L. japonica* polysaccharides are popularly used for treating throat diseases. The *L. japonica* lozenges have antibacterial, anti-inflammatory, and antitussive effects and can be used to treat bronchitis, pneumonia, and bronchiectasis. However, the complex structure of polysaccharides has hindered their extensive analysis, and the relationship between their structure and mechanism of action has not been clearly defined to date. The advances in polysaccharide chemistry and glycobiology will lead to the development of more polysaccharide-containing formulations that are beneficial for human health.

### 6.3. In the Daily Chemical Industry

With more research being conducted, *L. japonica* polysaccharides have potential applications in the daily chemical industry. They have good moisturizing, antioxidant, anti-allergic, anti-aging, and other properties and play a powerful role in daily chemical products. Yan et al. used the method of water extraction and alcohol precipitation to extract *L. japonica* polysaccharides. Combined with high-temperature fixation, hot air drying, grinding, high-pressure treatment, and sodium dodecyl sulfate, the extraction temperature and alcohol precipitation steps were reduced. The obtained *L. japonica* polysaccharides and sodium carboxymethyl fiber were combined in a 1:(3–4.5) mass ratio to form a compound system. The compound system can be used as a toothpaste thickener, which can not only improve the rheology of toothpaste and reduce the cost of toothpaste production but also reduce irritation in the oral cavity. At the same time, it can also be used as an additive component with good stability and safety, and also has anti-inflammatory, analgesic, and deodorant functions. In addition, a gargle solution containing *L. japonica* showed a potent inhibitory effect on oral pathogenic bacteria and viruses while maintaining the balance of a normal oral microbiota. It also prevented gum swelling, gum pain, and oral ulcers without causing any allergic reactions. Handmade soap made from vegetable oil and *L. japonica* polysaccharide can be used for makeup removal, bathing, and daily cleaning, and it can effectively remove skin stains, brighten the skin, and increase skin elasticity. These polysaccharides have good moisturizing and antioxidant effects because of their spatial structure and biological activity, which makes them effective components of anti-aging products. Various types of daily chemical products, including sunscreens, hand creams, masks, and body washes developed from *L. japonica* polysaccharides can protect the skin from UV rays, deeply hydrate the skin, and reduce oxidative damage. However, further studies are required on the compatibility and pharmacologic effects of *L. japonica* polysaccharides to develop safe and effective chemical products for daily use. Polysaccharides are green, natural, safe, and effective substances, which can be used to upgrade current products for the health-conscious population worldwide [100,101]. In conclusion, it is of profound significance in theory and practice to study the various effects of *L. japonica* polysaccharides, comprehensively develop and utilize the resources of *L. japonica*, continuously explore the value of *L. japonica,* and promote the development of the *L. japonica* industry, which will also bring huge economic benefits to the natural plant industry chain of *L. japonica*.

## 7. Conclusions and Perspectives

**Figure 5:**
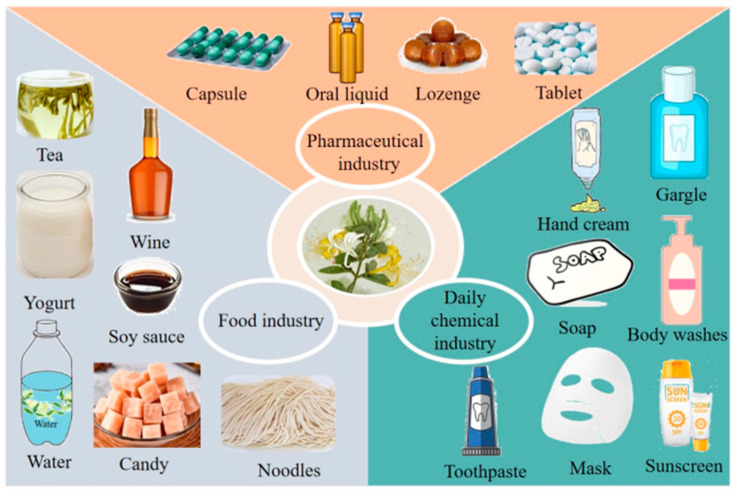
Practical and potential applications of *L. japonica* polysaccharides.

*L. japonica* is a natural plant with a long history of medicinal uses, and its small-molecule substances have been extensively explored. Currently, plant polysaccharides are being actively researched worldwide because of their potential therapeutic effects with minimal side effects. Polysaccharides extracted from *L. japonica* are an important class of biological macromolecular components, which are diverse, non-toxic, and safe. Their complex chemical structures and extensive biological activities have been evaluated in animal and cell line models. *L. japonica* polysaccharides have therapeutic properties, such as anti-diabetic, anti-inflammatory, anti-tumor, and antioxidant properties. Moreover, they can be used as stabilizers in dairy products, additives in pasta, sugar groups for brewing, and raw materials for cosmetics. The practical and potential applications of *L. japonica* polysaccharides are shown in Figure 5.

The extraction methods with optimal yields are a critical requirement for studying plant-derived polysaccharides. The existing extraction methods for *L. japonica* polysaccharides mainly include HWE, EAE, and UAEE. However, these methods are relatively simple, and there are some problems, such as having a low extraction purity, high purification cost, and complex process, so it is necessary to select the appropriate preparation technology to improve the yield of *L. japonica* polysaccharides. With the deepening of polysaccharide research and the progress of modern technology, several other extraction methods have also been used, such as microwave-assisted extraction (MAE) and supercritical fluid extraction (SFE). MAE is a newly developed technology that uses microwave energy for material extraction. The method uniformly heats the sample and is simple, fast, and efficient. *L. japonica* polysaccharides are mainly extracted from the initial flower or bud for medicine. When MAE is used, the double resistance posed by cell walls and intercellular substances can be easily overcome, and the polysaccharide components dissolve readily, thereby reducing the extraction time. SFE is a specific separation method using supercritical fluid as an extractant from liquids or solids, and the method has a high extraction efficiency with no solvent residue. This method does not require the purification step at later stages and is more convenient to use in the food and pharmaceutical industries. These separation methods can be used to obtain *L. japonica* polysaccharides efficiently and quickly for industrial applications.

To date, numerous studies have elaborated on the biological activities of *L. japonica* polysaccharides, and anti-diabetic, antioxidant, and anti-inflammatory activities have been actively researched. These polysaccharides decreased the blood glucose levels before and after meals, thereby providing a new research direction for the treatment of diabetes. *L. japonica* extract decreased acute and chronic inflammation caused by several bacteria, and the polysaccharides had a notable inhibitory effect on UC and RA. Further, *L. japonica* polysaccharides scavenged free radicals and protected the cells from oxidative damage, indicating their antioxidant potential in treating cardiovascular disorders and delaying the process of aging. Moreover, *L. japonica* polysaccharides expressed anti-depressant activity by modulating the NLRP3 pathway. The pharmacologic mechanism of its anti-depressant effect should be further explored as a potential treatment for depression.

However, to date, *L. japonica* as a medicinal resource has been underutilized. Traditionally, *L. japonica* polysaccharides are extracted from flower buds, which are picked early in the morning during the flowering period every year. The best quality buds are bluish-white and should be harvested and dried as soon as possible. However, the process is cumbersome with strict requirements. The global production of *L. japonica* flower buds is limited because of these constraints. Therefore, some scholars have studied the roots, stems, and leaves of *L. japonica* as sources of bioactive compounds. Polysaccharides can be obtained from the leaves and stem of *L. japonica*, and they have efficient biological activities. Therefore, the roots, stems, and leaves of the plant can also be used as raw materials for extracting the active components, including small-molecule compounds and macromolecular polysaccharides. However, further studies are required to analyze the potential of these raw materials. In addition, structurally modified polysaccharides can be used for the development of therapeutically effective *L. japonica* polysaccharides. The enhanced pharmacologic efficacy of polysaccharides through structural modifications will not only solve the problem of a shortage of resources but can also reduce the therapeutic doses of drugs. Overall, extensive use of *L. japonica* resources will promote the development of the *L. japonica* cultivation industry and boost the economy.

In this paper, the existing literature on *L. japonica* polysaccharide was reviewed, which laid a theoretical foundation for the in-depth study of *L. japonica* polysaccharides and is of great significance for promoting the development of *L. japonica* industry. It is hoped that more attention will be paid to *L, japonica* polysaccharides in the future, and a complete and mature analysis and identification technology will be established as soon as possible to realize the industrial production and application of polysaccharides.

## Figures and Tables

**Table 1:** A summary of extraction and purification methods of *L. japonica* polysaccharides.

Part	Extraction						Purification		Ref.
	Polysaccharide Fraction	Extraction Methods	Time (h/min)	Temperature (°C)	Solid–Liquid Ratio	Total Yield (%)	Polysaccharide Fraction	Purification Methods	
*L. japonica*	LJP-h	Water extraction	N/A	100 °C	N/A	N/A	LJP-N	DEAE-cellulose, Sepharose CL-6B column	[37]
*L. japonica*	LJP-h	Water extraction	N/A	100 °C	N/A	N/A	LJP-A	DEAE-cellulose, Sepharose CL-6B column	[37]
*L. japonica*	LJP-s	Water extraction	8 h	100 °C	1:20	N/A	N/A	N/A	[38]
*L. japonica*	LJP-b	Water extraction	8 h	100 °C	1:28	5.3%	LJP-N	DEAE-cellulose column, Sepharose CL-6B column	[39]
*L. japonica*	LJP-b	Water extraction	8 h	100 °C	1:28	5.3%	LJP-A-1	DEAE-cellulose column, Sepharose CL-6B column	[39]
*L. japonica*	LJP-b	Water extraction	8 h	100 °C	1:28	5.3%	LJP-A-2	DEAE-cellulose column, Sepharose CL-6B column	[39]
*L. japonica*	LJP-b	Water extraction	8 h	100 °C	1:28	5.3%	LJP-A-3	DEAE-cellulose column, Sepharose CL-6B column	[39]
*L. japonica*	LJP-b	Water extraction	8 h	100 °C	1:28	5.3%	LJP-A-4	DEAE-cellulose column, Sepharose CL-6B column	[39]
*L. japonica*	LJP-k	Enzyme-assisted extraction	50 min	45 °C	1:20	N/A	NA	N/A	[40]
*L. japonica*	LJP-z	Enzyme-assisted extraction	50 min	45 °C	1:20	N/A	N/A	N/A	[41]
*L. japonica*	LJP-d	Enzyme-assisted extraction	50 min	45 °C	1:20	N/A	N/A	N/A	[42]
*L. japonica*	CLJP-y	Water extraction	12 h	100 °C	1:20	4.32%	LJP-1-y	Macroporous adsorbent resin column D315 Macroporous adsorbent resin column D101, DEAE-cellulose 52	[43]
*L. japonica*	WLJP	Water extraction	6 h	100 °C	1:16	7.6%	WLJP-A0.2b	DEAE-cellulose column, Sepharose CL-6B column	[44]
*L. japonica*	CLJP-z	Water extraction	6 h	90 °C	1:30	5.4%	N/A	N/A	[45]
*L. japonica*	CLJP-z	Water extraction	6 h	90 °C	1:30	N/A	LJP-2-1	DEAE-cellulose column, Sepharose G-100 column	[45]
*L. japonica* flowers	LFA	Water extraction	16 h	N/A	N/A	3.60%	LFA03-a	DEAE-cellulose column, Sephacryl S-100 HR column	[21]
*L. japonica* flowers	LJ	Water extraction	24 h	N/A	1:20	4.6%	LJ-02-1	DEAE-cellulose 52 column, Sephacryl S-200HR column	[46]
*L. japonica* flowers	LJW	Water extraction	25 h	N/A	N/A	6.5%	LJW0F2	DEAE-cellulose column, Sephadex G150 column	[47]
*L. japonica* flowers	LJW	Water extraction.	25 h	N/A	N/A	N/A	LJW2F2	DEAE-cellulose column, Sephadex G150 column	[48]
*L. japonica* flowers	LJP-sd	Water extraction	16 h	N/A	N/A	5.5%	LJPB2	DEAE-cellulose 52 column, Sephacryl S-300 column	[22]
*L. japonica* flowers	Crude polysaccharide	Water extraction	3 h	80 °C	1:10	N/A	HEP-4	DEAE-52 cellulose column, Sephadex G-75 column	[49]
*L. japonica* flowers	LJP-bx	Water extraction	N/A	N/A	N/A	5.1%	WLJP-025p	DEAE-cellulose, Sepharose CL-6B column	[50]
*L. japonica* flowers	HP	Water extraction	6 h	100 °C	1:20	7.11%	HP-02	DEAE-cellulose 32 column, Sephacryl S-200HR column	[51]
*L. japonica* flowers	LJP-l	Water extraction	12 h	100 °C	1:20	5.1%	N/A	N/A	[52]
*L. japonica* flower buds	LJPs	Water extraction	9 h	80 °C	1:20	6.9%	LJP-w	DEAE-52 cellulose anion exchange chromatography column	[53]
*L. japonica* flower buds	CLJP-t	Water extraction	12 h	100 °C	1:20	4.7%	LJP-1-t	DEAE-cellulose column, Sephacryl S-300 column	[54]
*L. japonica* leaves	LJLP	Ultrasound-assisted enzymatic extraction	33 min	60 °C	1:20	14.76%	N/A	N/A	[55]
*L. japonica* caulis	LJCP	Water extraction	6 h	90 °C	1:30	1.8%	LJCP-2b	DEAE cellulose-52, Sephadex G75 column	[56]

**Table 2:** Source, compound name, molecular weights, monosaccharide composition, structures, and analytical techniques of *L. japonica* polysaccharides.

Source	Compound Name	Molecular Weights	Monosaccharide Composition	Structures	Analytical Techniques	Ref.
*L. japonica*	LJP-N	5.4 kDa	Glc:Gal:Ara = 43.7:25.1:31.2	LJP-N is a starch-like glucan with some arabinogalactan and/or arabinan domains.	N/A	[37]
*L. japonica*	LJP-A	400 kDa	GalA:Gal:Ara = 82.1:7.1:10.8	LJP-A is a pectic polysaccharide, mainly containing galacturonan with some galactan and/or arabinan domains.	N/A	[37]
*L. japonica*	LJP-s	18.5 kDa.	GalA:Rha:Gal:Ara:Glc:Xyl = 13.7:6.0:28.3:22.6:21.1:3.5	LJP-s may contain some RG-I pectin domains with galactan, arabinan, and arabinogalactan side chains, and some starch-like glucan domains.	HPLC, NMR	[38]
*L. japonica*	LJP-A-1	19.0 kDa	GalA:GlcA:Gal:Ara:Rha = 18.6:5.5:19.9:54.8:1.2	LJP-A-1 is mainly composed of Gal and Ara (>70%) with some GalA and GlcA residues.	HPLC, FT-IR, HPGPC	[39]
*L. japonica*	LJP-A-2	47.6 kDa	GalA:Gal:Ara:Rha = 53.8:17.4:26.4:1.1	LJP-A-2 is a HG domain-rich pectic polysaccharide, mainly composed of GalA (>50%) with some Gal and Ara residues.	HPLC, FT-IR, HPGPC	[39]
*L. japonica*	LJP-A-3	200.5 kDa	GalA:Gal:Ara:Rha = 55.4:11.5:31.9:1.2	LJP-A-3 is defined as a backbone mainly made up of (1→4)-linked α-D-GalpA, with a trace of→)4-α-D-GalpA-(1→2)-α-L-Rhap-(1→. The substituent were composed of (1→4)-linked Gal, (1→5)-linked Ara and (1→3,5)-linked Ara, which are substituted partly at C4 of Rha.	HPLC, FT-IR, HPGPC	[39]
*L. japonica*	LJP-A-4	383.8 kDa	GalA:Gal:Ara = 76.2:6.5:17.3	LJP-A-4 is an HG-domain-rich pectic polysaccharide mainly composed of GalA (>50%) with some Gal and Ara residues.	HPLC, FT-IR, HPGPC	[39]
*L. japonica*	LJP-k	N/A	Glu:Gal:Man:Rha:Xyl:Ara = 61.37:8.29:2.73:5.39:2.58:19.64	N/A	HPLC, FT-IR	[40]
*L. japonica*	LJP-z	N/A	N/A	N/A	FT-IR	[41]
*L. japonica*	LJP-d	N//A	Glu:Gal:Man:Rha:Xyl:Ara = 58.62:9.17:2.89:5.33:3.26:20.73	N/A	FT-IR, HPLC	[42]
*L. japonica*	LJP-1-y	17.5 kDa	GlcA:Glc:Gal:Ara:Xyl = 2.43:1:2.09:1.95:1.96	LJP-1-y has a spatial triple helix structure.	FT-IR, NMR, HPGPC, UV	[43]
*L. japonica*	WLJP-A0.2b	40.6 kDa	GalA:Rha:Gal:Ara:Glc:GlcA:Xyl:Man = 72.2:2.8:5.8:15.9:2.0:0.5:0.6:0.2	WLJP-A0.2b is shown to be dominated by HG domains and covalently linked with RG-I and RG-II domains. The RG-I domain contains α-L-1,5-arabinan; *β*-D-1,4-galactan; and AG-II side chains.	FT-IR, NMR, ESI-MS	[44]
*L. japonica*	CLJP	1450 kDa	Man:GluUA:GalUA:Glu:Gal:Ara:Rha:Fuc = 2.93:3.63:23.57:11.72:23.45:23.45:4.83:6.43	N/A	FT-IR, HPLC	[45]
*L. japonica*	LJP-2-1	1280 kDa	Man:GluUA:GalUA:Glu:Gal:Ara:Rha = 2.01:2.80:51.25:8.03:9.04:22.89:3.97	N/A	FT-IR, HPLC	[45]
*L. japonica* flowers	LFA03-a	67 kDa	Rha:Ara:Gal:GalA = 18.1:25.3:36.8:19.5	LFA03-a is proposed to have a backbone consisting of repeating unit →4)-α-D-GalpA-1-(1→2)-α-L-Rhap-(1→, with a substitution at C4 of rhamnose. The branches are mainly composed of T/1,5/1,3,5-linked Ara and T/1,4-/1,3/1,3,6-linked Gal.	GC-MS, FT-IR, NMR	[21]
*L. japonica* flowers	LJ-02-1	54 kDa	Rha:GalA:Gal:Ara = 10.77:7.88:15.45:65.89	LJ-02-1 is composed of a repeat unit of →4)-α-D-GalpA-1-(1→2)-α-L-Rhap-(1→ and is partly substituted at C4 of rhamnose. The branches contain T- and 1,4,6-linked *β*-D-Galp, T- and 1,5-linked α-L-Araf.	HPGPC, HPLC, GC-MS, NMR	[46]
*L. japonica* flowers	LJW0F2	37.1 kDa	glucose (99.7%)	LJW0F2 is elucidated to be an α-D-(1→4) glucan with an α-(1→4)-linked branch attached to the C6 position.	HPLC, FT-IR, NMR	[47]
*L. japonica* flowers	LJW2F2	7.2 kDa.	galacturonic acid (98.2%)	LJW2F2 is a homogalacturonan consisting of α-1,4-D-galacturonic acid.	FT-IR, NMR	[48]
*L. japonica* flowers	LJPB2	8.9 kDa	Ara:Man:Glc:Gal = 1.8:1.0:3.6:3.7	LJPB2 mainly contains 1, 4, 6-linked mannose; 1, 4-linked glucose; and 1, 4-linked galactose, with a highly branched structure of arabinan and terminal glucose.	GC-MS, FT-IR	[22]
*L. japonica* flowers	HEP-4	198 kDa	Man:Rha:GalA;Glc:Gal:Ara = 6.74:1.56:1.04:14.21:4.31:5.4	The backbone of HEP-4 is 1,4-*β*-D-Glcp; 1-α-D-Glcp, and other rhamnose residues branched at 1-*β*-D-Arap; 1,3,4-*β*-D-Arap; or 1,3,6-*β*-D-Manp.	HPLC, FT-IR, NMR	[56]
*L. japonica* flowers	WLJP-025p	23 kDa.	GalA:Gal:Ara:Glc:Man = 66.8:14.6:8.2:10.0:0.4	The primary structure of WLJP- 025p may be defined as an α-(1→4)-D-GalpA main chain, with *β*-(1→4)-galactan, α-(1→5)-linked, (1→3,5)-linked arabinan/arabinogalactan, and α-(1→4)-glucan side chains.	HPLC, UV, NMR	[50]
*L. japonica* flowers	HP-02	3.8 kDa	Ara:Rha:Man:Glc:Gal = 2.5:1.8:3.6:3.7:1.9	N/A	HPGPC, GC	[51]
*L. japonica* flowers	LJP-l	<1000 kDa	GalA:Rha:Gal:Ara:Glc:Man = 8.7:8.2:16.2:19.5:26.9:20.5	LJP-l is a heterogenous polysaccharide and may contain some of RG-I pectin domains.	HPLC, UV	[52]
*L. japonica* flower buds	LJP-w	N/A	Gal:Glc:Man:Rha:Xyl = 0.46:0.32:0.25:3.71:0.27.	N/A	GC	[53]
*L. japonica* flower buds	LJP-1-t	180 kDa	N/A	The main backbone chain of LJP-1-t is predominantly composed of Residue A and Residue B and is branched at O-3 position of Residue B with Residue C.	GC, FT-IR	[54]
*L. japonica* leaves	LJLP	N/A	Gal:Glc:Rib = 32.3:20.9:15.2	There are pyranose rings in the structure of LJLP polysaccharides.	FT-IR, HPLC	[55]
*L. japonica* caulis	LJCP-2b	7.0 kDa	Glc:GalA:Ara:Gal:Rha:Xyl:Man:GlcA = 3.89:2.49:1.87:1.51:1.00:0.40:0.21:0.19	LJCP-2b is α homogeneous heteropolysaccharide mainly composed of 1,3,6-*β*-D-Galp; 1,4-α-D-Glcp; 1,4,6-α-D-Glcp; 1,4-*β*-D-Galp; 1,2,4-α-L-Rhap; and 1,4-α-D-GalpA.	IR, NMR, ICS	[49]

**Table 3:** Biological activities of *L. japonica* polysaccharides and their underlying mechanisms of actions.

Biological Activities	Source	Polysaccharide Name	In Vitro or In Vivo	Indicated Concentrations	Models/Test System	Action or Mechanism	Ref.
Anti-diabetic effect	*L. japonica* flower buds	LJP-w	In vitro and in vivo	200, 400, and 800 mg/kg	α-Amylase and α-Glucosidase and male SD rats (200 ± 20 g)	In vitro: Inhibition of α-amylase with IC_50_ values of 61.2 ± 3.1 μg/mL and inhibition of α-glucosidase with IC_50_ values of 45.6 ± 1.9 μg/mL In vivo: ↓ TC, TG, LDL-C, VLDL-C, ALT, AST, and GGT *↑* HDL-C, CAT, SOD, and GSH *↑* Contents of liver and skeletal muscle glycogen *↑* Concentrations of hepatic pyruvate kinase and hexokinase	[53]
Anti-Alzheimer’s effect	*L. japonica* flowers	LFA03-a	In vitro	0.2 and 1 mg/mL	PC12 cells	Inhibition of A*β*42 aggregation and induction of neurite outgrowth	[21]
	*L. japonica* flowers	LJW0F2	in vitro	0.1, 1, and 10 g/mL	SH-SY5Y cells	Blockage of A*β*42 aggregation and reduction in its toxicity in SH-SY5Y cells	[47]
Anti-depression effect	*L. japonica* flowers	LJP-l	In vivo	30 and 100 mg/kg	Male KM mice (20 ± 2 g)	↓ NLRP3, IL-1*β*, and caspase-1 ↓ Immobility time *↑* Time spent of mice in the open arms and center zone	[52]
Antioxidant effect	*L. japonica* flowers	HEP-4	In vitro	200, 400, and 800 µg/mL	HepG2 cells	*↑* Scavenging effects on DPPH and ABTS radicals *↑* CAT and GSH-Px activity ↓ Levels of ROS and MDA	[49]
	*L. japonica*	WLJP-A0.2b	In vitro	0, 0.5, 1.0, 2.0, 5.0, and 10.0 mg/mL	HEK-293T cells	↓ Level of ROS *↑* Scavenging ability of ABTS, hydroxyl, and DPPH radicals	[44]
	*L. japonica* leaves	LJLP	In vitro and in vivo	100, 200, 400, and 800 mg/kg	Male nude KM mice	*↑* Effect on scavenging superoxide radicals *↑* Activities of CAT, SOD, GSH-Px, and TAOC in serum and liver ↓ Levels of MDA in serum and liver	[55]
	*L. japonica*	LJP-N, LJP-A-1, LJP-A-2, LJP-A-3 and LJP-A-4	In vitro	0, 0.25, 0.5, 1.0, 2.0, and 4.0 mg/mL	Erythrocyte	*↑* Scavenging ability of DPPH ABTS, hydroxyl, and superoxide radicals Inhibition of erythrocyte hemolysis Alleviation of oxidative stress	[39]
	*L. japonica*	CLJP and LJP-2-1	In vitro and in vivo	CLJP: 0, 100, 200, and 400 μg/mL LJP-2-1: 0 and 200 μg/mL	*C. Elegans* and Caco-2 cells	In vivo: ↓ Lipofuscin and MDA contents *↑* Sod-3, sod-2, gst-4, hsp-16.2, hsp-12.6, and hsp-60 levels *↑* SOD and CAT activities Promotion of the nuclear accumulation of DAF-16 Improvement of pharyngeal pumping and locomotion In vitro: Amelioration of the oxidative damage	[45]
	*L. japonica*	LJP-z	In vitro	10, 20, and 40 μg/mL	Male mice cardiomyocytes	*↑* Cardiomyocyte oxidative-stress survival rate *↑* Cardiomyocyte apoptosis rate ↓ AST, CPK, LDH, SOD, CAT, and GSH-Px activities ↓ ROS and MDA content ↓ Caspase-3, Caspase-8, and Caspase-9 activities	[41]
	*L. japonica* flowers	LJPB2	In vivo	50, 100, and 200 mg/kg/d	Male SD rats (200-250 g)	↓ MDA and NO levels *↑* SOD and GSH-Px activities *↑* Scavenging ability of DPPH	[22]
Immunoregulatory effect	*L. japonica*	LJP-N and LJP-A	In vivo	50 and 200 mg/kg	Female BALB/c mice (20–22 g)	*↑* Index of spleen and thymus *↑* Phagocytic function of macrophages *↑* Secretion of IL-2, IL-6, TNF-α, IgG, IgM *↑* Cytotoxic activity of NK cells	[37]
	*L. japonica*	LJP-d	In vivo	100 and 150 mg/kg	Male BALB/c mice (20.8 ± 2.3 g)	*↑* Index of spleen and thymus *↑* Phagocytic function of macrophages *↑* Levels of IL-2, TNF-α, and IFN-γ *↑* Percentage of CD4+ and CD8+ T cell subsets and ratio of CD4+/CD8+ *↑* Cytotoxic activity of NK cells	[42]
	*L. japonica* flowers	HP-02	In vitro and in vivo	250, 500, and 1000 μg/mL	Head kidney cells and common carp (*Cyprinus carpio* L.)	In vitro: *↑* Contents of TNF-α, IL-1*β*, IL-6, IL-12, IL-10, and TGF-*β* In vivo: *↑* Contents of TNF-α, IL-1*β*, IL-6, IL-10, and TGF-*β*	[51]
Anti-tumor effect	*L. japonica* flowers	LJ-02-1	In vitro	0.016, 0.031, 0.063, 0.125, 0.250, 0.500, and 1 mg/mL	BxPC-3 and PANC-1 pancreatic tumors cells	Inhibition rate of BxPC-3 was 66.7% Inhibition rate of PANC-1 was 52.1%	[46]
	*L. japonica* flowers	LJW2F2	In vitro	0.9, 1.8, 3.5, 7.0, and 13.9 µM	HMEC-1 cells	Inhibition of HMEC-1 cell tube formation and migration Disruption of EGFR/Raf/MEK/ERK and Dll4-Notch1 signaling axis	[48]
Anti-inflammatory effect	*L. japonica*	LJP-k	In vivo	50, 100, and 150 mg/kg	Male BALB/c mice	*↑* IL-2, TNF-α, IFN-γ, and SIgA concentrations *↑* Apoptosis rate of spleen lymphocytes Regulation of intestinal flora	[40]
	*L. japonica* Caulis	LJCP-2b	In vitro	50, 100, and 200 μg/mL	RA-FLS cells	↓ Cell viability, migration number, and adhesion capacity of RA-FLS cells ↓ Levels of IL-6 and IL-1*β* *↑* Apoptosis rate of RA-FLS cells	[56]
Anti-allergic effect	*L. japonica* flower buds	LJP-1-t	In vivo	20, 40, and 80 mg/kg	Female ICR strain mice (25–30 g)	↓ IgE, TNF-α, and histamine levels	[54]
	*L. japonica* flowers	WLJP-025p	In vivo and in vitro	In vivo: 30 and 60 mg/kg In vitro: 400 and 800 μg/mL	Female BALB/c mice (21 ± 2 g) and THP-1 cells	In vivo: ↓ Concentrations of IgE, IL-17 and IL-1*β* In vitro: Inhibition of the activation of NLRP3 inflammasome and inflammatory response	[50]
Anti-gouty arthritis effect	*L. japonica*	LJP-1-y	In vivo	100, 200, and 300 mg/kg	Male SD rats (200 ± 20 g)	↓ Uric acid level and XOD activity ↓ IL-1*β*, IL-6, TNF-α, and COX-2	[43]
Anti-alcohol-addiction effect	*L. japonica*	LJP-s	In vivo	50 and 100 mg/kg	Male KM mice (20 ± 2 g)	↓ Neurogenic damage ↓ p62, Glu, Glu/GABA, p-GluN2B, and p-VPS34 levels *↑* EAAT2, LAPM2, and LC3II levels Inhibition of reinstatement of CPP Inhibition of autophagy pathway activation *↑*	[38]

“↑” indicates increase; “↓” indicates decrease.
